# Infrared Spectroscopy as a Potential Diagnostic Tool for Medulloblastoma

**DOI:** 10.3390/molecules28052390

**Published:** 2023-03-05

**Authors:** Kornelia Łach, Aneta Kowal, Marta Perek-Polnik, Paweł Jakubczyk, Christopher J. Arthur, Wioletta Bal, Monika Drogosiewicz, Bożenna Dembowska-Bagińska, Wiesława Grajkowska, Józef Cebulski, Radosław Chaber

**Affiliations:** 1 Department of Pediatrics, Institute of Medical Sciences, University of Rzeszow, 35-310 Rzeszow, Poland; 2 Doctoral School, Institute of Medical Sciences, University of Rzeszow, 35-310 Rzeszow, Poland; 3 Department of Oncology, Children’s Memorial Health Institute, 04-730 Warsaw, Poland; 4 Faculty of Mathematics and Natural Sciences, University of Rzeszow, 35-310 Rzeszow, Poland; 5 School of Chemistry, University of Bristol, Bristol BS8 1TS, UK; 6 Clinic of Pediatric Oncology and Hematology, State Hospital 2 in Rzeszow, 35-301 Rzeszow, Poland; 7 Department of Pathology, The Children’s Memorial Health Institute, University of Warsaw, 04-730 Warsaw, Poland; 8 Center for Innovation and Transfer of Natural Sciences and Engineering Knowledge, University of Rzeszow, 35-310 Rzeszow, Poland

**Keywords:** medulloblastoma, MB, FTIR spectroscopy, Fourier transform infrared spectroscopy

## Abstract

**Highlights:**

**What are the main findings?**

- Comparison of healthy controls with subtypes of medulloblastoma using FTIR spectra.
- Analysis of physicochemical changes in FTIR spectrum in medulloblastoma and brain tissue.

**What is the implication of the main finding?**

- MB and normal brain tissue can be distinguished from one another to some extent using FTIR spectroscopy in the region 800–1800 cm^−1^.
- FTIR spectroscopy as a potential method in the diagnostics of medulloblastoma.

**Abstract:**

Introduction: Medulloblastoma (MB) is the most common malignant tumor of the central nervous system in childhood. FTIR spectroscopy provides a holistic view of the chemical composition of biological samples, including the detection of molecules such as nucleic acids, proteins, and lipids. This study evaluated the applicability of FTIR spectroscopy as a potential diagnostic tool for MB. Materials and methods: FTIR spectra of MB samples from 40 children (boys/girls: 31/9; age: median 7.8 years, range 1.5–21.5 years) treated in the Oncology Department of the Children’s Memorial Health Institute in Warsaw between 2010 and 2019 were analyzed. The control group consisted of normal brain tissue taken from four children diagnosed with causes other than cancer. Formalin-fixed and paraffin-embedded tissues were sectioned and used for FTIR spectroscopic analysis. The sections were examined in the mid-infrared range (800–3500 cm^−1^) by ATR-FTIR. Spectra were analysed using a combination of principal component analysis, hierarchical cluster analysis, and absorbance dynamics. Results: FTIR spectra in MB were significantly different from those of normal brain tissue. The most significant differences related to the range of nucleic acids and proteins in the region 800–1800 cm^−1^. Some major differences were also revealed in the quantification of protein conformations (α-helices, β-sheets, and others) in the amide I band, as well as in the absorbance dynamics in the 1714–1716 cm^−1^ range (nucleic acids). It was not, however, possible to clearly distinguish between the various histological subtypes of MB using FTIR spectroscopy. Conclusions: MB and normal brain tissue can be distinguished from one another to some extent using FTIR spectroscopy. As a result, it may be used as a further tool to hasten and enhance histological diagnosis.

## 1. Introduction

Medulloblastoma (MB) is an embryonal tumor that originates in the cerebellum. It is the leading cause of death from CNS malignancies, with an estimated 20% of all pediatric brain tumors being classified as MB [1,2]. The World Health Organization’s classification system distinguishes several histopathologic variants of MB, all of which are classified as stage IV tumors. In addition to classic MB, other variants include desmoplastic/nodular MB, MB with extensive nodularity (MBEN) and anaplastic and large cell MB [3]. Additionally, molecular profiling has shown a significant degree of genetic heterogeneity in MB, which has led to the identification of four major molecular subgroups: WNT, sonic hedgehog (SHH), Group 3, and Group 4. This genetic diversity presents a significant challenge for clinicians and each of these groups is associated with different age of disease onset and prognosis [4].

Fourier transform infrared (FTIR) spectroscopy is a powerful and versatile technique that allows researchers to shed light on the molecular composition of samples. Unlike traditional diagnostic tools, FTIR is non-destructive and does not require labeling, making it a rapid, cost-effective, and reproducible method for sample analysis. By measuring the characteristic absorbance peaks in the mid-infrared range (MIR, 400–4000 cm^−1^), FTIR can provide insights into the molecular makeup of a sample, including its levels of nucleic acids, proteins, carbohydrates, and lipids [5].

The use of FTIR has the potential to distinguish cancerous tissues from healthy tissues by analyzing the biochemical composition of samples at different stages of carcinogenesis. This technique can identify changes in peak position and quantitative changes of a given peak due to tumor progression, and has been used as a tumor prognostic marker. Statistical analysis of whole spectra can also help to determine characteristic patterns for specific tissue types, allowing for the accurate identification of samples of unknown status [6,7,8].

Our work, by investigating the biochemical changes associated with cancer progression, can provide valuable insights that may help to improve diagnosis and treatment for the medulloblastoma in the future by indicating the directions for further molecular research. In conclusion, through this work we hope to contribute to the field of cancer research and provide insights into the potential use of FTIR spectroscopy in the clinical setting.

## 2. Results

This study included fixed and paraffin-embedded tissues samples taken from 40 patients diagnosed with medulloblastoma.

### 2.1. Analysis of the Averaged FTIR Spectra

**Figure 1:**
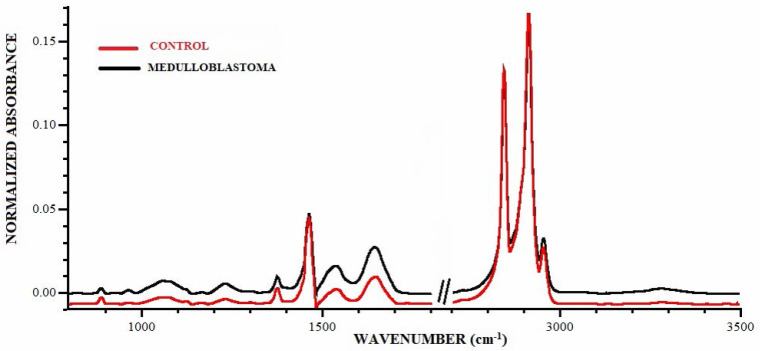
FTIR spectra of mean malignant cancer tissue of brain—medulloblastoma (black) and mean of control group (red). Measuring range 800–3500 cm^−1^.

The mean FTIR spectra for the tumor (MB) and control tissue are shown in Figure 1. Table 1 presents a summary of the absorbance values for peaks assigned to the major chemical groups based on histopathological subtype.

**Table 1:** Peak vibrational frequencies observed in the FTIR spectra of the different histological subtypes in FTIR spectra and their associated biochemical assignments [9,10,11,12,13].

Control	Classic Subtype	Desmoplastic Subtype	Anaplastic Subtype	Biochemical Assignment
889	889	888	889	C-C, C-O deoxyribose (carbohydrates, nucleic acids)
920	921	920	920	C-C residue a-helix, phosphodiester region (nucleic acids)
absent	964	964	964	C-C, C-O deoxyribose (nucleic acids)
1063	1060	1062	1062	C-O stretch, deoxyribose/ribosen (nucleic acids, lipids)
absent	1087	1085	1090	symmetric PO_2_ stretching (nucleic acids)
1125	absent	absent	absent	C-O, C-C, PO_4_ (carbohydrates, nucleic acids, RNA)
1168	1168	1168	1168	n(CC), d(COH), n(CO) stretching (lipids)
1233	1232	1233	1233	C-C stretch, C-H bend, C-O stretch, deoxyribose/ribose, DNA, RNA (nucleic acids)
1303	1302	1301	1301	deformation N-H cytosine (nucleic acids, lipids)
absent	absent	absent	1341	CH_3_ stretch symmetric (proteins)
1377	1377	1377	1377	mostly paraffin peaks, CH_3_ deformation, Amide III (lipids, protein)
1466	1466	1465	1465	mostly paraffin peaks, protein CH_2_ and CH_3_ bending of methyl, CH_2_ bending (lipids)
1541	1538	1539	1538	Amide II band mainly (N-H) bending and (C-N) stretching (proteins)
1650	1647	1646	1648	Amide I of proteins: stretching vibrations of the C-O (proteins) (In Table 2 is an assignment of secondary structure)
1737	absent	1737	1734	C-O vibrations (mostly lipids, proteins)
2847	2847	2847	2847	mostly paraffin peaks, symmetric stretching vibrations of CH_2_ (mostly lipids, proteins)
2915	2915	2915	2915	mostly paraffin peaks, asymmetric stretching vibrations of CH_2_ (mostly lipids, proteins)
2956	2956	2956	2956	mostly paraffin peaks, CH_3_ asymmetric stretching (mostly lipids, proteins)
3283	3283	3283	3282	stretching vibrations of NH groups in peptide chains and OH stretching of functional groups of water (protein, water)

**Table 2:** Assignment of amide I band position to secondary structure of protein.

Protein Secondary Structure	Controls (cm^−1^)	MB Classic Subtype (cm^−1^)	MB Large Cell/Anaplastic Subtype (cm^−1^)	MB Desmoplastic/Nodular Subtype (cm^−1^)
β-sheet	1631	1630	1630	1631
β-sheet	1641	1641	1641	1641
α-helix	1652	1649	1649	1648
α-helix	1658	1659	1659	1659
β-turn	1680	1679	1680	1680
β-sheet	1691	1691	1691	1691

Distinct differences in peak positions are observed between the averaged control and MB spectra for different histological subtypes. For example, the peak at 964 cm^−1^, corresponding to the C-C, C-O of deoxyribose in nucleic acids, is visible in the spectrum of distinct histological MB subtypes, while it is not present in the spectrum for the controls. Additionally, a shift in the symmetric PO_2_ stretching peak is observed in the MB specimens depending on their histological subtype: up to 1087 cm^−1^ for the classic, up to 1085 cm^−1^ for the desmoplastic, and up to 1090 cm^−1^ for the anaplastic subtype. This peak is not present in the spectrum of normal brain tissue.

A peak at 1125 cm^−1^ is observed in the control spectra, but not in any of the MB subtypes. This peak is likely related to changes in RNA [14]. Further research is needed to confirm the origin of this peak and its potential significance for the diagnosis and treatment of MB.

A distinctive peak is also observed at 1341 cm^−1^ (assigned to proteins) and it is present exclusively in the MB spectrum of the anaplastic subtype. The peaks assigned to proteins −1541 cm^−1^ (amide II) and 1650 cm^−1^ (amide I), are also observed in the MB spectra, but they are shifted to lower wavenumbers compared to the control spectrum. The peak at 1737 cm^−1^ corresponding to C-O vibrations (mostly lipid and proteins) is absent in the spectra of classical MB subtype, but is observed in the spectra of the controls and other MB subtypes.

### 2.2. PCA and HCA Analysis of the Chemical Profile

**Figure 2:**
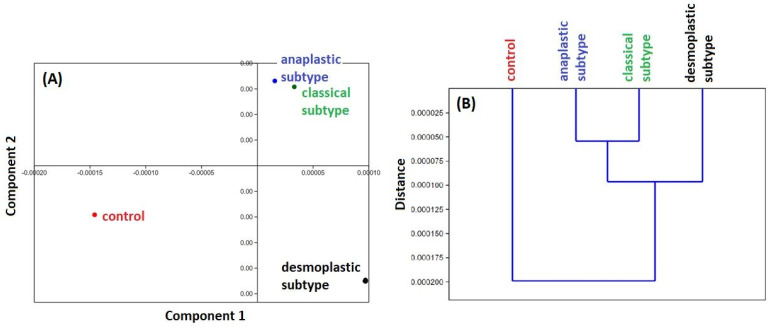
PCA (**A**) and HCA (**B**) analysis from ATR-FTIR spectroscopy of the controls and the three histological MB subtypes: (1) classic (green), (2) desmoplastic/nodular (black), (3) large cell/anaplastic (blue). Two-dimensional (2D) scores plot of samples with differences in biochemical components presented in fingerprint region. The analyses were performed on second derivative spectra.

Our principal component analysis (PCA) of the averaged second derivative spectra (shown in Figure 2A) reveals that the classic and anaplastic subtypes of MB have a different biochemical profile than the desmoplastic subtype and the controls. This is confirmed by our hierarchical cluster analysis (HCA) (shown in Figure 2B), which clearly distinguishes the biochemical profiles of the controls and MBs. Interestingly, the PCA and HCA also show that the spectra of anaplastic and classical MB subtypes cluster together, while the desmoplastic MB subtype and controls are clearly separated. These findings suggest that there are significant differences in the molecular makeup of the different subtypes of MB.

### 2.3. Protein Secondary Structure

FTIR is an established technique for the analysis of the secondary structure of proteins in which the C=O stretching-related absorptions are designated as amide I, whereas the N-H bending-related absorptions are termed amide II. The sensitivity of both the amide I and amide II bands to the secondary structure composition of a protein is due to the different hydrogen bonding patterns seen in the various secondary structure types. By analyzing these bands, it is possible to gain insight into the overall composition of protein secondary structures in a sample.

To do this, the region of the amide I protein between 1600 cm^−1^ and 1700 cm^−1^ was transformed into the second derivative and deconvoluted using a Gaussian function. Six peaks were identified in the amide I region, and their corresponding protein secondary structures are shown in Table 2.

**Figure 3:**
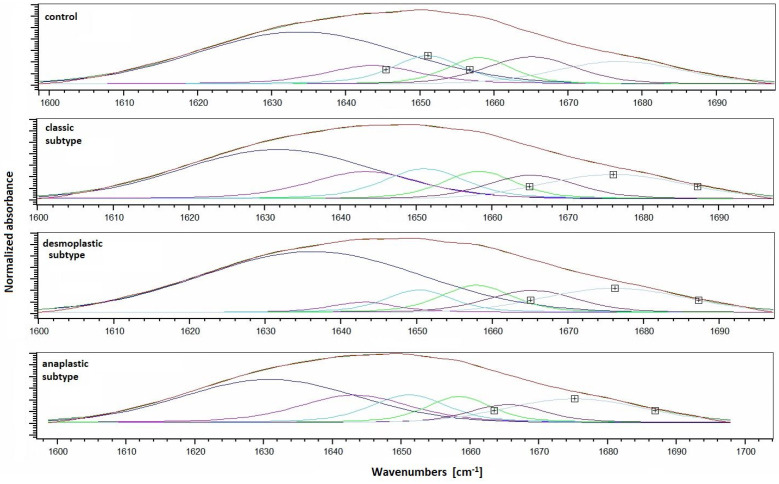
Curve-fitting of the amide I band of control and in various MB histological subtypes: classic, desmoplastic/nodular, and large cell/anaplastic.

The positions of the lines observed after deconvolution of amide I for the MB samples are very similar, with only a small shift (~1 cm^−1^) in the positions of the peaks. There is one notable exception to this trend, however, which is the peak located in the spectral range 1648–1659 cm^−1^, corresponding to the alpha-helical structure of the protein (Figure 3). In the control samples, this peak was located at 1649 cm^−1^, but in all MB subtypes, it was shifted to lower wave numbers. This indicates that there is a change in the alpha-helical composition of the proteins in MB samples compared to healthy tissue, which may be related to the underlying disease processes in MB.

**Figure 4:**
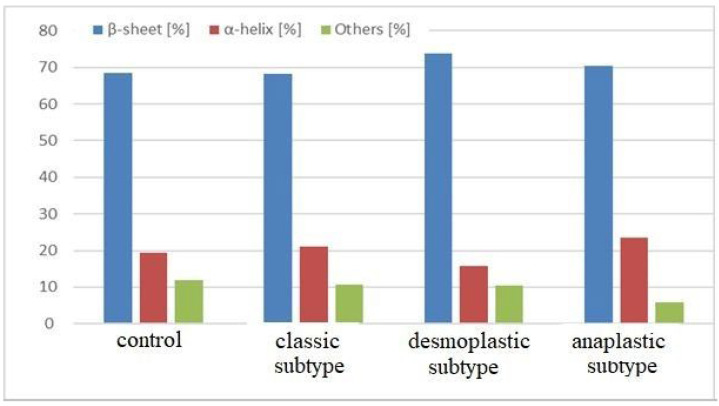
Protein secondary structure composition (%) of control and MB histological subtypes: classic, desmoplastic/nodular, and large cell/anaplastic.

Table 3 and Figure 4 show the percentage protein secondary structure composition of the samples calculated from the averaged control and MB spectra. The percentage changes in protein structure observed in these calculations are consistent with the results of the PCA and HCA analysis described earlier. These results indicate that there are significant differences in the protein secondary structures between the control and MB samples. In particular the anaplastic and desmoplastic subtypes show a striking change in protein composition.

### 2.4. Analysis of Absorbance Dynamics

**Figure 5:**
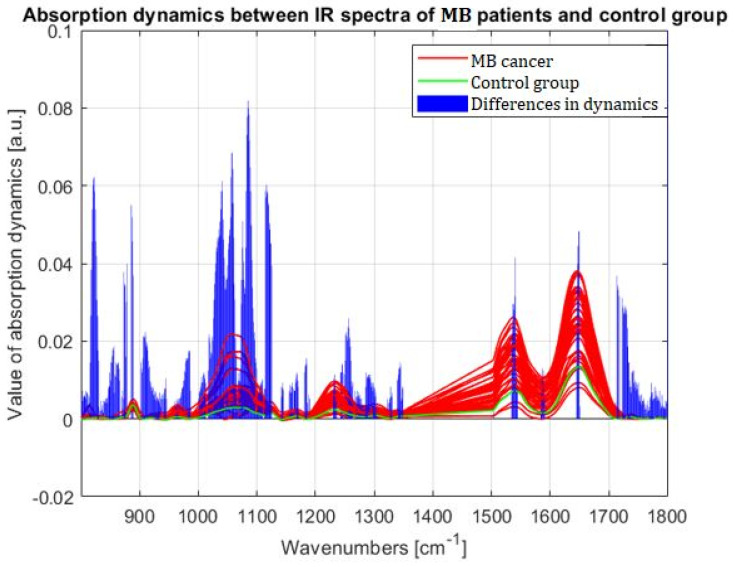
Graphical presentation of differences in absorption dynamics between the medulloblastoma patients and the control group in the region 800–1800 (cm^−1^) of wavenumbers (resected region of lipids 1350–1500 (cm^−1^)). The red line presents the spectra for medulloblastoma patients, the green the average for control group, and the blue vertical bars represent the summarized, normalized difference in absorbance dynamics between medulloblastoma patients and the control group.

In our previous work on COVID and leukemia, we demonstrated that differences in the first order derivative of spectra (rate of change of absorbance with wavenumber), termed absorbance dynamics, can be used to identify features in FTIR spectra that can differentiate patient groups. We applied this approach to the FTIR spectra recorded in this study and present the significant differences in absorption dynamics between the FTIR spectra of MB patients and the averaged spectrum of the control group in Figure 5. 

Using absorption dynamics, the probability of discrimination was determined for MB patients. The discrimination probability provides information on what percentage of the spectra of MB patients have significantly altered absorption dynamics in a specific wavenumber range compared to the averaged spectrum of the control group. The results of discrimination probabilities for the entire MB group and for each histological subtype are summarized in Table 4.

Although the absorption dynamics differentiate well between non-neoplastic control tissue and MB, the application of this method to differentiate between histological subtypes seems unachievable. Particularly noteworthy, however, is the 1714–1716 cm^−1^ range, in which the change in absorption dynamics allows 100% differentiation of control tissue from all MBs, regardless of their histological subtype.

## 3. Discussion

Rapid and accurate histopathological diagnosis of CNS lesions allows for the early use of appropriate treatments aiming to prevent disease development. If FTIR spectroscopy could detect substantial variations in the spectra of healthy and tumor tissue, it would improve the objectivity of histopathological examination and hasten diagnosis. It is necessary, therefore, that research into the biochemical profile of CNS tumors using vibrational spectroscopy is undertaken.

To date, researchers have documented several characteristics of FTIR spectra that can be used to discriminate between normal and neoplastic brain tissue.

An exploratory study by Gajjar et al. [15], examined several histologic types of primary tumors and metastatic brain tumors and demonstrated that ATR-FTIR or Raman spectroscopy could readily distinguish brain tumors from normal tissue. Unfortunately, no case of medulloblastoma was included in their study group. According to Gajjar et al., certain ratios of the peaks corresponding to major chemical groups have the potential to be used as a spectral biomarker for diagnosing brain tumors. The most significant differences were in the lipid/protein, phosphate/carbohydrate, RNA/DNA, and cholesterol esters/phenylalanine ratios. In a study by Depciuch et al. [10], the authors showed that signals from lipids, collagen, and proteins in Raman and FTIR spectra distinguished glioma and control brain tissues. Importantly, the authors concluded that lipids may be used as a spectroscopic marker for brain cancers. The areas pertaining to proteins and nucleotides were most appropriate in our investigation for distinguishing tumor and normal brain tissue. However, the lipid composition, which is characterized by strong C-O vibrational signals on FTIR spectroscopy, is possibly altered in the classical MB subtype. This can be seen by the absence of a peak at 1737 cm^−1^ in the spectra of classical MB samples, compared to the control group and other MB subtypes.

In our study, we also screened ratios of the most relevant chemical groups, but no significant differences were identified. The assessment of the concentrations of some molecular classes can be altered by the fixation and paraffining on the samples. Consequently, comparison of FTIR peak ratios can be biased and subject to error. In this study we therefore focused on analyzing the position of the most important peaks and their dynamics, especially in the spectrum fingerprinting region, rather than the calculation of their ratios. We believe this approach is more reliable and provides more objective results.

Hussein Ali et al. [11] showed that some discrimination between normal and cancerous tissue from the CNS could be achieved through differences in the protein (amide I and amide II) bands at 1650 and 1545 cm^−1^. This is consistent with our results, where the peaks assigned to proteins, 1541 cm^−1^ (amide II) and 1650 cm^−1^ (amide I), shift toward lower wave numbers in MB samples compared to control samples. Furthermore, Hussein Ali reported that the lipid bands at 2950–2850 cm^−1^ appeared weaker and were almost absent in tumor tissue. The authors concluded that ATR-FTIR may provide a new rapid method for the detection and classification of brain tumors. Regarding MB tissue, we obtained no differences in the lipid band at 2950–2850 cm^−1^ between tumor and control samples. It is important to note that the brain cancer samples studied by Hussein Ali et al. were not fully characterized and likely lacked examples of medulloblastoma.

Cameron et al. published an extensive study of 641 blood serum samples from brain cancer and control patients [16]. In their analysis, they were able to successfully differentiate several types of brain lesions (glioblastoma, meningioma, primary central nervous system lymphoma, and metastasis) with balanced accuracies >80%. Moreover, subtle differences in protein secondary structures between patient groups through Amide I deconvolution were revealed. Furthermore, Noreen et al. [17] demonstrated that diffuse and solid forms of glioma tumors can be discriminated by FTIR imaging based on molecular parameters and their secondary structure profile of the main collagen types found in the tissue. These studies highlight the potential of the secondary structure profiles as classification features. In our study directly on the fixed MB samples, some differences were shown in the amide I band corresponding to protein structures β-sheet and α-helix between controls and all three investigated MB subtypes (classic, large cell/anaplastic, and desmoplastic/nodular; Table 4). This distinction was subsequently confirmed in both the PCA and HCA analysis.

Previous studies have demonstrated the potential of FTIR spectroscopy for the diagnosis of various types of brain tumors, but to the best of our knowledge, none have specifically focused on medulloblastoma. Polis et al. [13] demonstrated that Raman spectroscopy can predict which tissue has tumorous biochemistry and can identify medulloblastoma. Raman spectroscopy makes use of the fact that tumors contain large amounts of protein and far less lipids, while healthy tissue is rich in both. Although FTIR and Raman spectroscopy both analyse molecular vibrational modes, unlike the Polis study, our quantitative analysis of the major chemical groups showed no significant differences [18].

In this study, we applied a novel analytical approach studying the absorption dynamics in the vibrational spectrum. We previously applied this method in studies on acute lymphoblastic leukemia in children [7] and SARS-CoV-2 infection in pregnant women [19]. Control brain tissue and MB were successfully differentiated using this method; however, it does not appear to be possible to distinguish between histologic subtypes of MB. The band 1714–1716 cm^−1^ seemed to be the most significant for accurately distinguishing MB and control samples. Each MB patient had altered dynamics in this band compared to the average spectrum of the control group. This peak is assigned to the carbonyl of thymine or guanine [20] and may indicate changes in nucleic acid composition, which was also proposed by Dovbeshko et al. [21].

We applied several methods to distinguish the spectra of histopathological subtypes of MB and normal brain tissue by FTIR spectroscopy. Although the spectra of MB and normal brain tissue have visually observable variations, the differences in the chemical composition of the particular histological subtypes are too small to definitively differentiate them to individual subtypes. Although it was possible to classify the control and MB spectra into three classes (control, classical/anaplastic, desmoplastic) by PCA and HCA analysis, it was not possible to assign each individual sample to a specific histological subtype.

It is worth noting that there are some limitations to this study that affect our findings. A major limitation lies in the fact that ATR-FTIR spectroscopy is an ex vivo technique and can only be performed on fixed tissue [15]. Thus, FTIR spectroscopy cannot be used as a tool to define MB tumor boundaries during surgery, but it can potentially be used for objectifying and accelerating histopathologic examination of these tumors. Second, our sample size is limited due to the rarity of medulloblastoma and the lack of normal, living brain tissue (for understandable reasons) to be able to fully assess the value of FTIR spectroscopy. Despite this we believe our study still provides valuable insights on the potential value of vibrational spectroscopy for the diagnosis of brain cancers. Finally, our analysis was performed on fixed and paraffin-embedded tissue samples, which can alter their chemical composition. However, even with these changes, a holistic analysis of the spectra should permit the different classes of samples to be distinguished based on their overall spectral patterns [22,23].

## 4. Materials and Methods

### 4.1. Characteristics of the Studied Population

Formalin-fixed paraffin-embedded (FFPE) samples from forty patients (with a median of age 7.8 years; range 1.5–21.5 years, 31 boys and 9 girls) diagnosed with medulloblastoma between 2010 and 2019 were included in the study. All patients were treated in the Department of Oncology, The Children’s Memorial Health Institute IPCZD in Warsaw, Poland. Their characteristics are presented in Table 5. A histopathologist specializing in juvenile neurooncology validated the tumor’s histologic subtype in each case. The control group consisted of normal brain tissue obtained from four children who had been diagnosed with causes other than cancer. Table 1 shows the clinical features of the patients.

### 4.2. Preparation of Samples

Tissue sections (10 μm thick) were obtained from FFPE blocks by microtome sectioning and mounted on low-E, IR reflective, CaF_2_ slides (Crystran) slides for ATR-FTIR spectroscopy. Samples were applied to slides in triplicate. Deparaffining was not performed in order to preserve as much information as possible from the basic chemical structure of the tumor samples analyzed. The spectrum analysis accounted for this by removing bands corresponding to the methylene groups, of which paraffin is primarily composed, to differentiate between different types of spectra.

### 4.3. Infrared Spectrometer—Characteristics, Measurement Parameters

A Bruker Vertex 70v spectrometer equipped with an attenuated total reflection (ATR) attachment and diamond crystal was used to measure tissue sections in the mid-infrared range (800–3500 cm^−1^, excluding 1800–2800 cm^−1^). Spectra were recorded in triplicate with a spectral resolution of 2 cm^−1^, using 32 scans. In the case of paraffin-saturated samples, air was monitored as the background.

### 4.4. Data Analysis

The recorded spectra were vector normalized and the baseline corrected using a concave rubber band correction, with 64 base points. The average wave number values for each functional group were calculated to compare their intensities for each type of tissue being studied. Hierarchical cluster analysis (HCA) was performed to obtain information about the similarity between samples and to exclude outliers. Spectral distances were calculated as Euclidean distance, and the individual clusters were extracted according to the unweighted pair group method (UPGMA). In addition, principal component analysis (PCA) was performed to obtain information about the variability of the spectra across samples. These two procedures were performed on the second derivative in fingerprint regions between 800 cm^−1^ and 1800 cm^−1^ (excluding the range for paraffin 1350–1500 cm^−1^ to avoid data bias).

To analyze the secondary structure of the proteins in samples, first the region from 1600 cm^−1^ to 1700 cm^−1^ of amide I of the protein was transformed into a second derivative allowing the position of the component bands to be identified [24,25,26]. The minima of the second derivative indicates the positions of the individual spectral lines that can be assigned to a specific protein conformation [18,27,28]. Gaussian deconvolution (WiRE 5.3) of the FTIR spectra can then be performed to identify the contributions from each secondary structure type.

Finally, we performed an analysis of the absorbance dynamics between histological subtypes of MB. To do this, we first identified the wavenumber regions with different absorption dynamics between the spectra of different subtypes. We then used these regions to distinguish the MB spectra based on their unique absorption dynamics.

### 4.5. Software

Data and statistical analysis were performed using the following software: OPUS 8,5 (Bruker Optik GmbH 2019, Ettlingen, Germany), WiRE 5.3 (Renishaw, Gloucestershire, UK), Past software 4.04 (Oslo, Norway), and KnowItAll Academic Edition (JohnWiley&Sons, Inc., version 2018, Hoboken, NJ, USA). 

## 5. Conclusions

ATR-FTIR spectroscopy has the potential to improve the histopathological analysis of CNS tumors by providing a quick and objective method for distinguishing healthy and tumor tissue. Previous studies have shown that FTIR spectra can be used to differentiate various types of tumors and even subtypes within the same tumor type. Our study adds to this body of research by demonstrating that FTIR spectroscopy can be used to differentiate medulloblastoma from healthy brain tissue. Furthermore, we have shown that the positions of key peaks in the FTIR spectrum, particularly those corresponding to proteins and nucleotides, can provide valuable information about the underlying molecular changes associated with medulloblastoma. The differences in the chemical compositions of the different medulloblastoma subtypes are, however, insufficient to allow them to be distinguished by FTIR alone. Although more research is needed in this area, our findings add to the growing body of literature showing the potential for FTIR spectroscopy as a useful tool for the study of brain tumor biochemistry.

## Figures and Tables

**Table 3:** Percentage composition of secondary structure in each histological subtype, according to relative area of component bands.

	β-Sheet (%)	α-Helix (%)	Others (%)
control	68.5	19.5	12.0
classic subtype	68.2	21.2	10.6
desmoplastic/nodular subtype	73.7	15.8	10.5
large cell/anaplastic subtype	70.5	23.6	5.9

**Table 4:** Regions of wavenumbers, which can be used as potential markers to discriminate medulloblastoma patients by IR spectrum with discrimination probability. Values > 80% are shown in bold.

Wavenumbers (cm^−1^)	Discrimination Probability (%) for Patients with Medulloblastoma (All Genotypes)	Discrimination Probability (%) for Genotype 1 (Classic Subtype)	Discrimination Probability (%) for Genotype 2 (Desmoplastic/Nodular)	Discrimination Probability (%) for Genotype 4 (Large Cell/Anaplastic Subtype)
829–830	69–95	76–95	60–100	61–92
873–875	77–90	76–90	**80**	69–92
920–932	59–77	48–81	20–100	38–84
944–945	69–87	71–95	60–100	69
1063–1065	72–79	**81–86**	60–80	61–69
1115–1125	74–95	**81–100**	**80–100**	61–84
1285–1287	77–87	71–81	**100**	76–92
1343–1345	**82–90**	**81–90**	**100**	76–92
1540–1541	69–90	**81–86**	40–80	62–100
1649–1650	**85–97**	**85–100**	**100**	76–92
1714–1716	**100**	**100**	**100**	**100**
1723–1736	64–97	71–100	40–100	46–92

**Table 5:** Clinicopathological characteristics of patients.

Male/Female Ratio	31/9
age: median (range)	7.8 (1.5–21.5)
histological subtype	
classic	21
desmoplastic/nodular	5
anaplastic/large cells	14
molecular subtype	
WNT	3
SHH	4
other	33

## Data Availability

Not applicable.

## Sample Availability

The data presented in this study are available on request from the corresponding author.
